# First In Vivo Applicational Data of Foam-Based Intrathoracic Chemotherapy (FBiTC) in a Swine Model

**DOI:** 10.3390/ph17010045

**Published:** 2023-12-27

**Authors:** Carolina Khosrawipour, Jakub Nicpoń, Zdzisław Kiełbowicz, Przemysław Prządka, Bartłomiej Liszka, Kacper Zielinski, Veria Khosrawipour, Shiri Li, Hien Lau, Joanna Kulas, Agata Diakun, Wojciech Kielan, Agata Mikolajczk-Martinez, Mariusz Chabowski

**Affiliations:** 1 Faculty of Medicine, Wroclaw Medical University, 50-345 Wroclaw, Poland; 2 Department of Surgery, Faculty of Veterinary Medicine, Wroclaw University of Environmental and Life Sciences, 50-366 Wroclaw, Poland; 3 Clinical Department of Anaesthesiology and Intensive Care Unit, Wroclaw Medical University, 50-367 Wroclaw, Poland; 4 Department of Surgery, Petrus-Hospital Wuppertal, 42283 Wuppertal, Germany; 5 Division of Colon and Rectal Surgery, Department of Surgery, New York Presbyterian Hospital, Weill-Cornell College of Medicine, New York, NY 10065, USA; 6 Department of Surgery, University of California-Irvine (UCI), Irvine, CA 92697, USA; 7 Faculty of Veterinary Medicine, Wroclaw University of Environmental and Life Sciences, 50-375 Wroclaw, Poland; 8 2nd Department of General Surgery and Surgical Oncology, Wroclaw Medical University, 50-556 Wroclaw, Poland; 9 Department of Biochemistry and Molecular Biology, Faculty of Veterinary Medicine, Wroclaw University of Environmental and Life Sciences, 50-375 Wroclaw, Poland; 10 Faculty of Medicine, University of Science and Technology Wroclaw, 58-376 Wroclaw, Poland; 11 Department of Surgery, 4th Military Hospital, 50-981 Wroclaw, Poland

**Keywords:** intrathoracic chemotherapy, doxorubicin, malignant pleural effusion, pleural metastasis, foam-based drug application

## Abstract

Background: For decades, both intraperitoneal and pleural chemotherapy (IPC) have been delivered as a liquid solution. Recent studies suggest that foam carriers outperform liquid carriers for locoregional chemotherapy. For the first time, this study aims to evaluate the feasibility, safety, and characteristics of foam-based intrathoracic chemotherapy (FBiTC) in an in vivo setting. Methods: In this study, contrast-enhanced FBiTC with doxorubicin was delivered via video-assisted thoracoscopy (VAT) in three swine under general anesthesia. Intraoperative and postoperative parameters, blood analyses, vital signs, and anesthesiologic data were collected. Additionally, an intraoperative computer tomography (CT) scan was performed, and histological tissue sections were collected and further analyzed using fluorescence microscopy. Results: FBiTC was delivered without major complications. End-tidal capnometry detected increased CO_2_ levels with reduced peripheral oxygen saturation and increased blood pressure and heart rate. No major intra- or postoperative complications were observed. CT scans confirmed a multidirectional distribution pattern of foam. Postoperative laboratory workup did not reveal any critical changes in hemoglobin, white blood count, or platelets. There was no evidence of critical kidney impairment or liver function. Fluorescence microscopy of tissue specimen detected doxorubicin in pleural tissues. Discussion: Our preliminary results are encouraging and indicate that FBiTC is feasible. However, to consider a possible clinical application, further studies are required to investigate the pharmacologic, pharmacodynamic, and physical properties of FBiTC and to ensure the safety of the overall procedure regarding oxygenation levels and capnography parameters.

## 1. Introduction

After decades of clinical and experimental research, the treatment of peritoneal and pleural surface malignancies remains challenging due to an overall poor prognosis. In the case of peritoneal metastasis, median survival rates are only a few months, while pleural metastasis (PM) prognosis is only slightly better [1,2,3]. PM is associated with a variety of tumor entities that may spread within the pleural cavity [4,5]. Currently, different disciplines can be involved in the management of patients who suffer from PM. These often include different specialists, such as oncologists, pulmonologists, surgeons, and anesthesiologists. Different concepts are applied in both the management of PM and associated malignant pleural effusion (MPE). Some of these include the placement of catheters to release excessive MPE. Additionally, some catheters can also be used for liquid chemotherapy instillations or combined with surgical procedures, such as pleurectomy and talc poudrage [6,7]. Recently, pressurized intrathoracic aerosol chemotherapy (PITAC) was established as a novel concept to directly target PM [8]. More specifically, PITAC delivers a highly concentrated chemotherapeutic solution into the pleural cavity via aerosol generation. Due to promising clinical results in MPE patients, PITAC has gained worldwide attraction in treating PM and MPE [8]. Locoregional chemo aerosolization [9] allows for the enhancement of drug concentration without increasing the volume of the carrier solutions. This technology intends to simultaneously improve drug distribution [10,11] and retain high local drug concentrations [12]. The application of PITAC requires an operating room, an aerosol-creating device, a high-pressure injector, and specifically trained surgical personnel. Despite a wide range of improvements, we have also observed limitations with the use of pressurized aerosol chemotherapeutic applications regarding drug distribution inhomogeneities and chemotherapeutic fluid accumulation [13]. Despite these shortcomings, the scientific community has realized the potential of cancer management via locoregional anticancer drug instillations in various forms. Significant clinical research has focused on improving the outcomes of pleural and peritoneal surface malignancy treatments, especially via hyperthermic intraperitoneal chemotherapy (HIPEC) and cytoreductive surgery (CRS) or pressurized intraperitoneal aerosol chemotherapy (PIPAC). In contrast to the classic approach of fluid-based chemotherapy instillations or the aerosolization of chemotherapeutic solutions, a novel concept of foam-based locoregional chemotherapy has been recently proposed [14] by Schubert et al. The authors describe a variety of potential benefits of the use of a foam carrier. In fact, the foam has some qualities that overcome the limitations of aerosol and liquid applications. Foam expansion is different than liquids, and it has been proven to have a higher drug-carrying capacity than gas. In foam, a low total drug dosage can be combined with a high drug volume while still maintaining a high drug concentration; this is possible because more than 95% of the actual drug volume consists of air [14]. To evaluate whether foam-based intrathoracic chemotherapy (FBiTC) is feasible for PM, we investigated its feasibility, safety, and behavior in an experimental in vivo swine model. This study is the first to gain in vivo data on some major characteristics. These include surgical and anesthesiologic parameters as well as foam behavior within the thoracic cavity.

## 2. Results

### 2.1. Clinical Evaluation

No major intra- or postoperative complications were observed. FBiTC was successfully applied into the right hemithorax of all three swine via the 10 mm trocar. No complications were witnessed during extubation. All animal behavior was within the norms and did not indicate elevated stress levels throughout the 7th day of postoperative mentoring.

### 2.2. Evaluation of CT Scan

**Figure 1:**
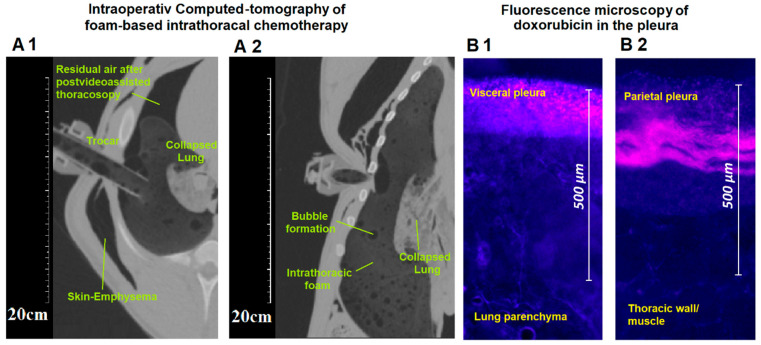
(**Left side**): computed tomography of the thorax during foam-based intrathoracic chemotherapy. (**A1**) Transversal and (**B1**) longitudinal section with visualization of the collapsed lung, the intrathoracic foam, and the placed trocar. (**Right side**): demonstration of fluorescence microscopy of (**A2**) visceral and (**B2**) parietal pleura with a marked depth level of doxorubicin in the demonstrated section.

The delivered FBiTC was detected in a CT scan (Figure 1A). Horizontal, vertical, and transversal foam expansion was noticed; residual air volume after the VAT was detected in the upper ventral thoracal cavity. The CT scan did not indicate large areas of intrathoracic foam degradation. No basal liquid was detected at the time of the scan. A limited number of larger “bubbles” were detected. The inner diameter of the insufflation tube did not cause foam degradation. The scan revealed the collapse of the right lung, as well as iatrogenic-caused emphysema in one of the three swine.

### 2.3. Evaluation of Vital Parameters

**Figure 2:**
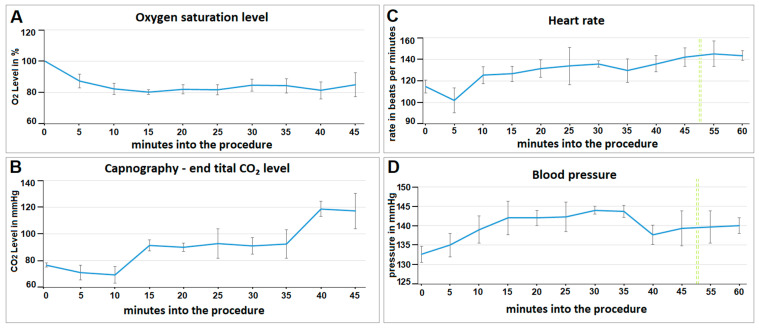
Intraoperative and early postoperative vital parameters of video-assisted-thoracoscopy (VAT) with foam-based intrathoracic chemotherapy in vivo. (**A**) Oxygen saturation during 45 min of surgery. (**B**) Capnography with end-tidal CO_2_ levels. (**C**,**D**) Intraoperative and early postoperative (after extubation), vital parameters: (**C**) heart rate and (**D**) blood pressure. The extubation phase is indicated past the green line.

Intraoperative oxygen saturation decreased during surgery with insufflation of the foam-based intrathoracic chemotherapy. After an initial drop to around 80% oxygen saturation levels, the measurements stabilized and slightly increased again towards the end of the procedure (Figure 2A). End-tidal capnography slightly decreased in the first 5 to 10 min and then rapidly increased after 15 min (Figure 2B). End-tidal capnography CO_2_ plateaued at around 90 mmHg for a while and then increased toward the end of the procedure at around 40 min. Within the first 5 min, the heart rate (beats per minute/bpm) dropped from 115 to 102 bpm (Figure 2C). Then, the heart rate constantly increased and stabilized at around 140 bpm. The blood pressure increased for the first 15 min during the procedure (Figure 2D). It plateaued at around 140 mmHg after 15 min. The heart rate dropped slightly at the beginning of the procedure and stabilized again towards the end.

### 2.4. Evaluation of Postoperative Laboratory Parameters

**Figure 3:**
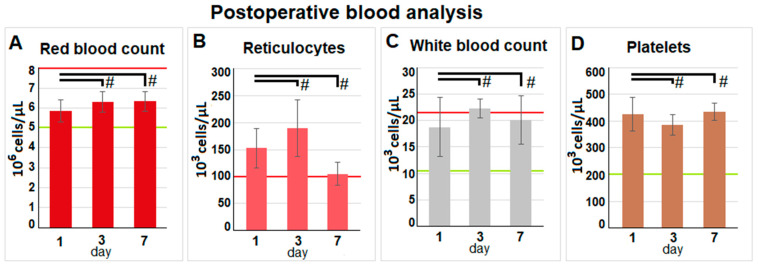
Blood was collected on the 1st, 3rd, and 7th days after surgery. Listed are (**A**) red blood count, (**B**) reticulocytes, (**C**) white blood count, and (**D**) platelet count. The mean values are presented by the height of the columns; the standard deviations are indicated. Red and green lines show normal reference levels (95% interval—red: upper limit and green: lower limit) for the parameters. Significance levels are indicated by # with *p* > 0.05.

The postoperative blood analyses revealed a stable red blood cell count, a stable white blood cell count, and a stable platelet count (Figure 3A,C,D).

**Figure 4:**
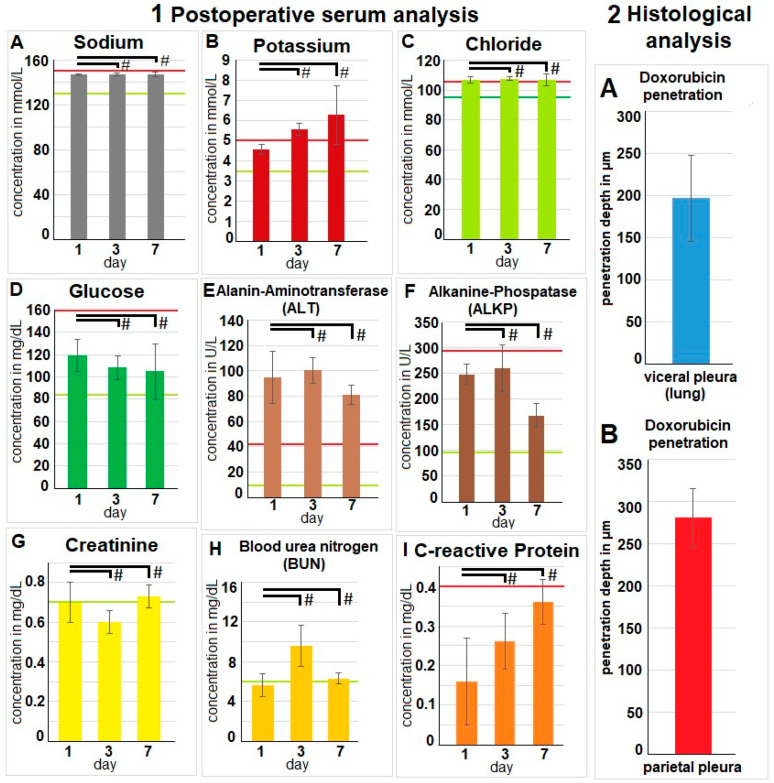
(**Left side 1**): Blood was collected after surgery on the 1st, 3rd, and 7th day. Electrolytes are indicated in (**A**) sodium, (**B**) potassium, and (**C**) chloride. (**D**) Glucose, (**E**) alanine aminotransferase, (**F**) alkaline phosphatase, (**G**) creatinine, (**H**) blood urea nitrogen, and (**I**) C-reactive protein. The mean values are presented by the height of the columns, and the standard deviation is added; # indicates that no statistical difference was detected. Green lines indicate the lower reference level, and red lines indicate the upper reference level for each parameter. (**Right side 2**): Data of fluorescence microscopy of (**A**) visceral and (**B**) parietal pleura with mean depth level of doxorubicin and standard deviation are listed. Significance levels are indicated by # with *p* > 0.05.

There were no indications of infection, bleeding, or any other relevant loss of blood volume or red blood cells. An insignificant increase in reticulocytes was observed on day three (Figure 3B). Postoperative serum analyses were extensive, and most parameters were within physiological parameters. Analyses revealed stable sodium levels (Figure 4(1A)). Chloride levels were slightly increased. Glucose levels did not reveal any significant changes. Potassium levels were elevated, and a slight increase in C-reactive Protein (CRP) levels was noted. However, mean CRP levels remained below 0.4 mg/dL. While the liver enzyme alanine aminotransferase (ALT) increased beyond the physiological threshold, alkaline phosphatase remained within physiological levels. No indication of kidney impairment was detected with physiological blood urea nitrogen (BUN) and creatinine levels.

### 2.5. Fluorescence Histology

The swine tissue samples were retrieved after the autopsy. As described, samples were removed from different pleural and visceral pleura locations. During the fluorescent microscopy, we could confirm that the tissue samples were exposed to doxorubicin. The mean tissue penetration rate detected by fluorescence histology was 196 ± 51 µm for the visceral pleura. For the parietal pleural, the depth was higher at 280 ± 35 µm (Figure 4(2A,B)).

## 3. Discussion

Currently, extensive research efforts are being conducted to improve treatments for pleural and peritoneal surface malignancies. While most studies still focus on conventional fluid instillations, new concepts that extend beyond the traditional therapies and technologies have been introduced. These concepts include new drug particles to cover pleural and peritoneal surfaces and the application of hyperthermia and dehydration to enhance antitumoral effects [15]. After some initial in vitro testing, this current study presents the first in vivo data for FBiTC. Our results suggest that FBiTC application in the surgical setting is feasible. Foam delivery and expansion are promising, with an overall good distribution and broad coverage, including most of the pleural cavity. 

Currently, there are no quantitative data available on the total amount of chemotherapy in the pleural tissue for FBiTC. There are multiple options to evaluate the chemotherapeutic levels in the pleura. These include high-performance liquid chromatography, as well as fluorescence intensity measurement of the exposed tissue. These options must be meticulously analyzed and standardized to ensure reliability and viability. Additionally, doxorubicin has fluorescence qualities, which are beneficial and could be used. However, its autoxidative properties make it unclear how reliable doxorubicin is. Doxorubicin also suffers from rapid photochemical degradation, which impairs its fluorescence qualities to a significant degree. 

The foam is relatively stable and does not rapidly degrade. Thus, no local pockets of untreated areas or fluid accumulation were observed. At this point, we should mention that the foam was not removed after intrathoracic delivery. The concept does not include or consider foam removal. The foam spontaneously collapses and is then gradually absorbed by the surrounding tissue.

Postoperative lab results are encouraging and show no indications of short-term complications, including organ failure or infections. Moreover, autopsy results did not display any relevant macroscopical changes or complications, including surface adhesion or atelectatic lung areas. With respect to intraoperative aspects, our data suggest that vital parameters remain within physiologic levels, indicating that FBiTC application is both safe and feasible. One limiting aspect that should be mentioned is that we did not take tissue samples from all organs to evaluate that there was no significant tissue damage. However, the results from the blood workup do not support the idea of any organ failure at a distant site. 

The collapse of one side of the lung and the application of a bicarbonate-based delivery system lead to a variety of changes in blood pressure, oxygenation, and expiratory CO_2_ levels. Some of these changes may persist until the end of the procedure; this might be a challenge during the extubation process, as well as during the clinical setting regarding the patient’s general condition. However, nowadays, VAT is a standard procedure and is certainly manageable in most cases of patients who are not limited by their overall pulmonary function. 

Nevertheless, whether the administered amount of bicarbonate changes the blood bicarbonate levels to a critical point that may restrict the ability for FBiTC therapy should be further studied. There are data showing the effects of citrate administration regarding pharmacokinetics, toxicity and side effects [16,17]. The observed side effects are mostly related to hypocalcemia or a disbalance in the potassium levels that are due to pH level [18,19,20,21]. Currently, a study is planned that focuses on the tolerance level of administered intrathoracic and intraperitoneal bicarbonate. This study was performed to evaluate if FBiTC is a feasible concept. It is important to note that this study is not comparative. A comparison against lavage or any other concept could be part of a follow-up study. At this point, it was important to evaluate whether the concept of FBiTC should be studied further.

Different types of foam-based drug delivery systems have been studied. One important aspect of such concepts is the characteristics of the foam and its pharmacological properties. Another important aspect is whether the foam can be applicable at the desired location; this means that the technical application in the particular location should be possible. Therefore, any limitations that may occur on the skin are due to the pharmacological nature of the foam. Yet, there may be critical physical limitations regarding the foam application in the internal cavity. The internal cavities, including the intraabdominal, intrathoracic, and colorectal cavity, experience limitations that are not present when dealing with the external body surface areas; this could be one reason the application of foam-based drug delivery systems is already well established for a variety of dermatological diseases. Some of these foams carry corticosteroids as a therapeutic ingredient [22,23,24]. However, the foam can also carry other drug classes, such as antibiotics [25]. There is some clinical experience with foam-based applications in vaginal delivery [23] or colorectal delivery [24]. Nevertheless, the application of foam in larger closed cavities has barely been examined. One unique application of foam delivery is sclerotherapy. Nonetheless, these sclerotherapeutic injections were performed in a very limited area, and a low volume of foam was applied [26].

The criteria to perform a safe video-assisted thoracoscopy are established. Although we did not observe severe complications, we cannot distinguish if the observed cardiovascular and gasometric changes are due to pulmonary collapse and not because of the bicarbonate carrier system. The effects of thoracoscopy on oxygen saturation, carbon dioxide levels, and the cardiovascular system have been described [27]. Thoracoscopy impacts these systems, and various factors influence patients’ therapeutic compliance. These factors include the length and invasiveness of the thoracoscopy, the patient’s general condition, and overall lung capacity. All these factors impact how well the patient can cope with the stress of a thoracoscopic procedure. Additionally, the overshooting of intrathoracic pressure during thoracoscopy has been described as a significant factor, especially regarding adverse effects on the cardiovascular system [28,29].

Moreover, the extent of side effects of the bicarbonate carrier system remains unknown. Therefore, further studies are required to explore the pH, cardiovascular, and gasometric influences and the gradual foam collapse of FBiTC. Due to the few swine used during this study, the ability to conduct a thorough investigation is limited.

## 4. Materials and Methods

### 4.1. Sequence of Procedures

This study used an in vivo swine model, as it is the closest comparable model to that of a human adult chest, excluding various primate models. The animals were not genetically altered. There were no recorded genetic defects or any type of immunological deficits.

All animals were examined by a veterinary doctor before recruitment. The included animals did not show any signs of distress or disease. This study included a total of three Polish large white breed pigs (local supplier, Zerniki Wielkie).

**Figure 5:**
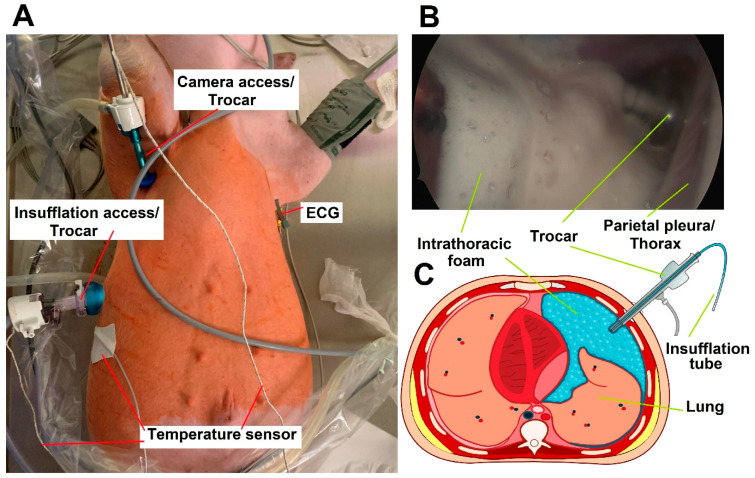
(**A**). Video-assisted thoracoscopy (VAT) with foam-based intrathoracic chemotherapy (FBiTC) in vivo. (**B**). Intraoperative and thoracic view during foam insufflation during the in vivo experiment. (**C**). Section model of foam-based intrathoracic chemotherapy.

Their weight was approximately 50 kg; they were female and 65 days old. Following surgical preparation and intubation, each swine received a video-assisted thoracoscopy (VAT) on the right hemithorax (Figure 5A). Contrast-enhanced FBiTC with doxorubicin was introduced (Figure 5B) in the right thoracic cavity, and computed tomography (CT) of the thorax was performed. The last swine received an additional dosage of foam-based intraperitoneal chemotherapy; this was carried out to evaluate the safety of intraperitoneal delivery for a separate follow-up study on foam-based intraperitoneal chemotherapy (FBIC). The swine were monitored after the VAT for 7 postoperative days until their euthanization on the last day. Following euthanization, the left hemithorax was filled with FBiTC. Both hemithoraces were then opened. The right hemithorax was inspected, while tissue samples were removed from the left hemithorax according to established protocols.

### 4.2. Video-Assisted Thoracoscopic (VAT) for FBiTC Application in an In Vivo Swine Model

All swine received adequate care. The management of the swine was in compliance with the 8th edition of the Guide for the Care and Use of Laboratory Animals published by the National Institutes of Health [30]. For premedication, an injection of midazolam (0.3 mg/kg, WZF Polfa S.A., Warsaw, Poland) combined with medetomidine (0.02 mg/kg, Cepetor 1 mg/mL, CP-Pharma Handelsgesellschaft, Burgdorf, Germany) and ketamine (9 mg/kg, Ketamina 100 mg/mL, Biowet Puławy sp. z o.o., Puławy, Poland) was performed. Propofol was given at 1 mg/kg for analgetic considerations. After initial intubation and preparation for surgery, anesthesia was maintained with isoflurane 1%. Fentanyl (2 g/kg) was used as an additional analgesic. An intravenous line was maintained by a constant infusion of crystalloid fluid at 0.2–0.3 µg/kg/min. VAT was prepared with the swine in the supine position. A trocar (10 mm, Kii^®^Balloon Blunt Tip System, Applied Medical, Rancho Santa Margarita, CA, USA) was inserted through the 6th Intercostal space. Another smaller trocar (5 mm) was inserted at the 2nd to 3rd Intercostal space for the insertion of the endoscopic camera (Figure 5A). The right-sided thoracic cavity was insufflated with CO_2_ (Olympus UHI-3 insufflator, Olympus medical life science and industrial divisions, Olympus, Shinjuku, Tokyo, Japan). A diagnostic overview was performed via video-assisted thoracoscopic (VAT). The imaging system was provided by Karl Storz (camera systems/Tuttlingen, Germany). Under visual monitoring, the “foam-insufflation” tube of the foam-generating system was introduced, and the FBiTC was delivered. The correct placement of the tube was confirmed via visual imaging. FBiTC was started, and the camera was slowly removed (Figure 5B). After delivery of the foam, the inflow tubes were removed, and the intubated swine was placed onto a CT table in the neighboring room. The CT scan was performed, and the swine was placed back on the operating table. Parallel to the FBiTC, an intraoperative thoracic CT scan was performed (Figure 1(A1,A2)).

### 4.3. Postoperative Monitoring

One operative procedure was performed per day. All swine were housed together and monitored for behavior changes, feeding habits, indication of pain, and surgical site infection for a total of 7 postoperative days. Blood samples were drawn for blood cell, platelet, and serum parameter analysis on postoperative days 1, 3, and 7 (1 d, 3 d, 7 d). On the last postoperative day (7 d), an autopsy was performed.

### 4.4. Euthanization

Midazolam (0.1 mg/kg, Midanium 5 mg/mL), medetomidine (0.02 mg/kg, Cepetor 1 mg/mL) and ketamine (8 mg/kg, Ketamina 100 mg/mL) were given prior to euthanization. The recommendations for euthanization were followed [30] according to the guidelines. Intravenous sodium pentobarbital (50 mg/kg) and Morbital (12 mg/kg) were used. 

FBiTC was immediately repeated postmortem to remove adequate tissue samples from all desired locations. The contralateral site was chosen to make sure that there was no prior intervention in the cavity that could potentially affect penetration rates.

FBiTC with doxorubicin was delivered into the left hemithorax according to the in vivo protocol. Visceral and parietal pleural tissue samples were retrieved for later microscopic fluorescent analysis. A median thoracotomy was performed, and both lung cavities were thoroughly examined. Tissue samples were removed from multiple sites of the left pleura.

### 4.5. Microscopic Analysis of the Pleural Tissue

After removal of the tissue samples from the thorax cavity, they were rinsed with sterile NaCl 0.9% solution. Thereafter, all samples were frozen in liquid nitrogen. Four tissue samples were removed from the visceral pleura, and another four tissue samples were removed from the parietal pleura. These samples reflected the upper, lower, ventral, and dorsal locations of the lung and the intrathoracic cavity. These samples provided a reasonable distribution of various pleural locations. The prepared cryosections (7 µm) were then mounted with VectaShield containing 1.5 µg/mL 4′,6-diamidino-2-phenylindole (ProLong^®^ Gold DAPI, Thermo Fisher Scientific, Waltham, MA, USA). Doxorubicin penetration into the tissue was measured using a Nikon Eclipse 80i (Nikon Instruments Europe B.V., Amsterdam, The Netherlands). The software that was applied was NIS-Elements V2.3 (Nikon Instruments Europe B.V., Amsterdam, The Netherlands). The light source was a D-LEDI Fluorescence LED Illumination system (Nikon) with filter cubes for C-LED385 for DAPI and C-LED525 for doxorubicin. The innermost positive staining for doxorubicin accumulation (vs. pleural surface) was measured in micrometers (Figure 1B) to evaluate the penetration depth.

### 4.6. The Bicarbonate-Based Foam Carrier

The ratio of foam ingredients for the bicarbonate foam was mathematically and experimentally predetermined. The basic chemical reactions were analyzed and quantified to determine the most suitable molar and pH combination. The foam is based on a combination of citric acid (Sigma–Aldrich, St. Louis, MO, USA) and sodium bicarbonate (Sigma–Aldrich, St. Louis, MO, USA). The chemotherapeutic agent that was added to the foam was doxorubicin hydrochloride (PFS^®^, 2 mg/mL, Pfizer, Sandwich, UK). In our model, this corresponds to a total dosage of 3 mg of doxorubicin, a common dosage used in PITAC/PIPAC. 

The liquid citric acid solution and the liquid bicarbonate solution containing the doxorubicin are continuously injected into a sealed 200 mL reaction chamber. The reaction chamber is connected to a tube system. From there, it redirects all the foam created towards the exiting tube that was previously inserted via trocars into the abdomen. As the foam in the reaction chamber expands, it pushes more foam into the abdominal cavity. The foam production and delivery rate can be controlled and maintained by the speed at which the ingredients were injected into the reaction chamber. 

The doxorubicin load capacity is 50.0–52.0 mg/mL. The solution of the foam carrier is fluid. This solution is added to a bicarbonate solution with doxorubicin. On impact, both of these solutions will create a foam. The mean foam density is 0.083 air/fluid with a peak of 0.048 air/fluid at 6.5 min. The foam generation process is endothermic with a mean peak delta of 4.7 degrees Celsius at 2 min. The mean pH is at about 7.6 pHs ± 0.3. The half-life of the foam-based carrier is 12 min ± 2 min, and the in vivo drug elimination pattern differs for each compound. For the CT scan, an iodide-based contract media was also added (AccupaqueTM 350 mg J/mL, GE Healthcare, Chicago, IL, USA). 

### 4.7. Environmental Control and Housing Standards

The recommendations set by the guidelines [30] were followed for animal housing and environmental control. The animals were kept indoors, and the environmental temperature was stable at around 18–25 °C before and after the surgical procedures. The relative humidity was around 40% to 60%. The appropriate ventilation of the facilities was ensured. However, no bacterial filter was installed because it was not necessary for this study. The animals were not genetically altered. There were no recorded genetic defects or any type of immunological deficits. Appropriate indoor illumination was ensured, and diffuse lighting was diffused throughout the holding area. A regular diurnal cycle was maintained. Noise and vibration in the holding area were kept to a minimum. All animals had an adequate amount of space available. Bedding and nesting material straw was provided. The straw was removed and replaced every other day for sanitary purposes. The swine received environmental enrichment strategies that prevented undesirable, overly aggressive behavior and provided stress relief for the animals. The animals were kept within a fasting period of 5 h before the operation. Postoperatively, all animals had unrestricted access to food and water. All animals were examined by a veterinary surgeon before and after surgery. Afterward, swine were kept in an individual holding area until they were fully awake. A daily routine check-up was made every day, including the weekend. 

### 4.8. Statistical Data Analyses 

A Kruskal–Wallis test (non-parametric test) was performed to evaluate if any significance levels were detected between the groups. GraphPad Prism (GraphPad Software Inc., San Diego, CA, USA, version 8.0.2) was used to analyze the laboratory blood and serum parameters. The statistical analysis included the following parameters: mean, median, and percentiles. The probability levels were accordingly indicated in the figures and figure legends with * *p* < 0.05 ** *p* < 0.005, and # *p* > 0.05; a *p*-value below 0.05 was considered statistically significant.

### 4.9. Ethical Approval and Regulations

The Local Board on Animal Welfare approved the experiments (UCHWALA NR 029/2021/P1). All procedures went according to Polish regulations and European Union laws.

### 4.10. Graphic Design

The used graphic programs were Inkscape 1.0.1, 2020, and GNU, USA. The programs were supported by Microsoft Windows Office 2019.

## 5. Conclusions

Our data indicate that FBiTC could be a feasible option to treat PM and MPE. However, further research is required to assess the full potential of this application, especially regarding its intrathoracic use. Prior to this study, no in vivo data were available for foam-based applications in PM treatment. Although this study is preliminary and preclinical, it provides some important insight into the potential of FBiTC to improve PM treatment. It encourages further studies to evaluate this novel concept. However, further research on this topic is needed, and more data and parameters must be analyzed. Therefore, this study provides valuable first in vivo data to help establish further studies. Aspects like efficacy, safety, and biodistribution are only some parameters that would be of interest in follow-up studies.

## Data Availability

Data are contained within the article.
